# Effectiveness of a Hospital-Based Work Support Intervention for Female Cancer Patients – A Multi-Centre Randomised Controlled Trial

**DOI:** 10.1371/journal.pone.0063271

**Published:** 2013-05-22

**Authors:** Sietske J. Tamminga, Jos H. A. M. Verbeek, Monique M. E. M. Bos, Guus Fons, Jos J. E. M. Kitzen, Peter W. Plaisier, Monique H. W. Frings-Dresen, Angela G. E. M. de Boer

**Affiliations:** 1 Coronel Institute of Occupational Health, Academic Medical Center, University of Amsterdam, Amsterdam, the Netherlands; 2 Finnish Institute of Occupational Health, Kuopio, Finland; 3 Department of Internal Medicine, Reinier de Graaf Groep, Delft, the Netherlands; 4 Department of Gynaecology, Academic Medical Center, University of Amsterdam, Amsterdam, the Netherlands; 5 Department of Internal Medicine, Albert Schweitzer Hospital, Dordrecht, the Netherlands; 6 Department of Surgery, Albert Schweitzer Hospital, Dordrecht, the Netherlands; Davidoff Center, Israel

## Abstract

**Objective:**

One key aspect of cancer survivorship is return-to-work. Unfortunately, many cancer survivors face problems upon their return-to-work. For that reason, we developed a hospital-based work support intervention aimed at enhancing return-to-work. We studied effectiveness of the intervention compared to usual care for female cancer patients in a multi-centre randomised controlled trial.

**Methods:**

Breast and gynaecological cancer patients who were treated with curative intent and had paid work were randomised to the intervention group (n = 65) or control group (n = 68). The intervention involved patient education and support at the hospital and improvement of communication between treating and occupational physicians. In addition, we asked patient's occupational physician to organise a meeting with the patient and the supervisor to make a concrete gradual return-to-work plan. Outcomes at 12 months of follow-up included rate and time until return-to-work (full or partial), quality of life, work ability, work functioning, and lost productivity costs. Time until return-to-work was analyzed with Kaplan-Meier survival analysis.

**Results:**

Return-to-work rates were 86% and 83% (p = 0.6) for the intervention group and control group when excluding 8 patients who died or with a life expectancy of months at follow-up. Median time from initial sick leave to partial return-to-work was 194 days (range 14–435) versus 192 days (range 82–465) (p = 0.90) with a hazard ratio of 1.03 (95% CI 0.64–1.6). Quality of life and work ability improved statistically over time but did not differ statistically between groups. Work functioning and costs did not differ statistically between groups.

**Conclusion:**

The intervention was easily implemented into usual psycho-oncological care and showed high return-to-work rates. We failed to show any differences between groups on return-to-work outcomes and quality of life scores. Further research is needed to study which aspects of the intervention are useful and which elements need improvement.

**Trial Registration:**

Nederlands Trial Register (NTR) 1658

## Introduction

In recent years, advances in cancer screening and cancer treatment have improved the survival rates for patients with cancer. An increasing number of cancer patients are therefore able to live many years beyond the original cancer diagnosis and face new challenges upon cancer survivorship. For cancer patients of working age, returning to work is a key aspect of survivorship because it is often experienced as an important part of their recovery [1]. Furthermore, work contributes to personal, social, and economic well-being, and therefore return-to-work is associated with the quality of life of cancer patients [2]–[4].

Unfortunately, not all cancer patients are able to return-to-work and many of these patients have more adverse work outcomes in comparison to the general population. For instance, the risk of unemployment is estimated to be 37% higher for cancer patients compared to non-cancer controls [5] and return-to-work rates are estimated to vary between 30–93% depending on for instance cancer type and treatment [6], [7]. Furthermore, a portion of cancer patients face a decrease in income [8] and suffer from impaired work functioning compared to the general population [9], [10]. Finally, the employer and the society at large are also affected due to the costs of absenteeism, disability pension, and loss of productivity [11].

Intervention studies aimed at enhancing the return-to-work of cancer patients are rare, especially randomised controlled trials [12], [13]. However, we developed an intervention based on previous studies that demonstrated effective results for enhancing the return-to-work of cancer patients [12], and we developed this intervention together with various stakeholders involved in the return-to-work process of cancer patients [14]. An early intervention – meaning soon after diagnosis or early in treatment - is most appropriate because the longer the duration of sick leave, the more difficult return-to-work is to achieve [15]. For delivering an early intervention, a hospital-based intervention is most appropriate, as most cancer patients do not have contact with their employer or occupational physician during early phases of their cancer treatment and their advice seems to be influential [16], [17]. In addition, previous studies have shown that early interventions could be most effective [12]. Furthermore, return-to-work should be part of the complete psycho-oncological care package and should not be dealt with in isolation [18].

Our hypothesis is that a hospital-based intervention will enhance the return-to-work of cancer patients, as work is not typically addressed at the hospital [19]. Furthermore, an important and modifiable prognostic factor for the return-to-work of cancer patients is self-assessed work ability which varies according to treatment type and cancer diagnosis [7]. Self-assessed work ability may readily be improved by providing patient education and support that addresses misconceptions concerning return-to-work [20]. The objective of this study was to determine the effect of a hospital-based work support intervention for cancer patients on return-to-work and quality of life, which was achieved.

## Materials and Methods

The medical ethics committee of the Academic Medical Center approved the study, and the medical ethics committees of the six participating hospital advised positively regarding feasibility of the study. Patients signed informed consent forms prior to participation in the study. This trial was registered at the Dutch National Registry:NTR1658 (http://www.trialregister.nl/trialreg/admin/rctview.asp?TC=1658).

Both the design of the study and the content of the hospital-based work support intervention have been described in detail elsewhere [14]. We used items from the CONSORT statement for improving the quality of reporting randomised trials [21]. The protocol for this trial and supporting CONSORT checklist is available as supporting information; see Checklist S1 and Protocol S1.

### Patients

Cancer patients between 18 and 60 years of age who had been treated with curative intent at one of the six participating hospital departments, had paid work, and who were on sick leave were eligible to participate. Treatment with curative intent was defined as an expected 1-year survival rate of approximately 80%. We excluded patients who were not sufficiently able to speak, read, or write Dutch, had a severe mental disorder or other severe co-morbidity, and for whom the primary diagnosis of cancer had been made more than two months previously. We monitored non-response by assessing the proportion of patients who participated in comparison to all eligible patients.

### Hospital-based work support intervention

The hospital-based work support intervention began a few weeks after the onset of the study and was spread across a maximum of 14 months. The hospital-based work support intervention consisted of the following components: 1) delivering patient education and support at the hospital, as part of usual psycho-oncology care; 2) improving communication between the treating physician and the occupational physician; and 3) drawing-up a concrete and gradual return-to-work plan in collaboration with the cancer patient, the occupational physician, and the employer [14]. We integrated patient education and support regarding return-to-work into the usual psycho-oncological care in the form of 4 meetings that lasted 15 minutes each. This care was delivered by an oncology nurse or medical social worker (hereafter referred to as nurse). In addition, a least one letter was sent to the occupational physician to enhance communication. We also asked the occupational physicians to organise a meeting between the patient and the employer to draw-up a return-to-work plan. The key aspects of the hospital-based work support intervention included the patient education and support at the hospital and the provision of information to the occupational physician. In the Netherlands, patients must provide their consent to allow medical information to be sent from a treating physician to an occupational physician. Therefore, we were only able to inform the occupational physicians of patients who provided this form of consent.

### Study design

This study was designed as a multi-centre randomised controlled trial with a follow-up period of two years. Here we report the results of the first follow-up year. Eight departments from six hospitals in the Netherlands participated in the study.

The treating physician or nurse informed the cancer patients of the study a few weeks after their diagnosis and determined patient eligibility by assessing the inclusion and exclusion criteria. The research team contacted patients who were eligible and willing to participate and enrolled these patients in the study. After the patients had filled in the baseline questionnaire, one of us [ST] allocated the eligible patients to the intervention or to the control group using the computerised randomisation programme ALEA [22]. The allocation ratio was set as equal in the programme. Stratified randomisation was applied for two important prognostic factors for return-to-work [23]; age (<50 or ≥50) and cancer diagnosis (i.e. hospital department). Minimisation was applied to equalise group sizes. The patient date of each consecutive patient were entered in the programme and according to the conditions mentioned above the programme randomly assigned the patients to the intervention or the control group. The allocation was irrevocable and was not changed during the study nor during the analysis. Patients and providers were immediately informed of the allocation as it was impossible to conceal allocation for this intervention.

Questionnaires were administrated to the patients at baseline and at 6 and 12 months of follow-up. The follow-up questionnaires were mailed to the patients' homes with a postage-paid envelope enclosed. Both the questionnaire data and the information from the nurses who delivered the intervention were gathered for the economic evaluation. Outcome measures and cancer treatment were assessed at all time points. Socio-demographic factors and prognostic factors for time until return-to-work were assessed at baseline only.

### Measurements

The primary outcomes were return-to-work and quality of life. The intervention was considered effective if patients in the intervention group had a significantly shorter time to return-to-work (in days) than did patients in the control group, provided that their quality of life had not significantly deteriorated.

Return-to-work was measured both as the rate of return-to-work at one year of follow-up and as the number of calendar days between the first day of sick leave and the first day at work (either part-time or full-time) that was sustained for at least 4 weeks. Quality of life was assessed with the Short Form-36 (SF-36) [24], which included all subscales and a Visual Analogue Scale (VAS). Secondary outcomes included work ability, work functioning, and costs. Work ability was assessed using the first question of the Work Ability Index (WAI) [25]. Impaired work functioning was assessed with the Work Limitation Questionnaire (WLQ) [26], which could only be filled in if a patient had (partly) returned to work.

We conducted the economic evaluation from a societal perspective. We included lost productivity costs and work adjustments costs for both groups and costs to deliver the intervention for the intervention group. Productivity loss was determined by multiplying the cumulative net number of hours on sick leave by the estimated price of productivity loss based on age and gender [27]. We assumed that when a patient partially returned to work, his/her productivity was 100% during the hours of partial work resumption. We calculated productivity losses using both the human capital approach and the friction costs approach [27]. For the human capital approach, all hours on sick leave were included for 100%. For the friction costs approach, all hours on sick leave with a maximum of 167 days were included for 80% [27]. Costs to deliver the intervention were determined by combining the training costs and the costs to deliver the intervention. Training costs consisted of trainer costs, study material costs, and attendance costs for the nurses. Costs to deliver the intervention consisted of the mean hour of investment multiplied by the average nurse wage and subsequently multiplied by 42% overhead costs [27], and the mean hour of investment of the secretary for sending of the letters to the occupational physician, as well as the printing costs for the information leaflet. As the letter from the treating physician to the occupational physician was a copy of the letter to the general practitioner, no additional costs for the treating physician to produce these letters were taken into account.

The socio-demographic factors measured at baseline included the number of days between the first day of sick leave and enrolment in the study, marital status, time since diagnosis, breadwinner status, position at work, shift work, years in current position, years of paid employment, income, importance of work (VAS), and company size.

Prognostic factors for time to return-to-work of the cancer patients included [7], [23] age, gender, education, diagnosis, cancer treatment, number of working hours according to contract, physical workload (Questionnaire of Perception and Judgement of Work (VBBA)) [28], fatigue (Multidimensional Fatigue Inventory (MFI)) [29], depression (Centre for Epidemiologic Studies for Depression Scale (CES-D)) [30], co-morbidity, self-efficacy (general self-efficacy scale (ALCOS)) [31], and clinical characteristics (i.e. diagnosis and treatment).

### Sample size

The calculation of the patient sample size was based on two earlier studies focused on return-to-work in cancer patients [23], [32]. Based on the return-to-work rates in these studies, we assumed a relative risk of not returning to work of 0.53 for individuals in the intervention group versus those receiving usual care [14]. With a power of 80% and two-sided significance level of p<0.05, the sample size required was 109 patients in each group [33]. Assuming that 20% of the initial patients would be lost to follow-up, 270 patients should have been recruited to gather 246 patients at 12 months of follow-up. To account for at least 10% missing data at baseline, 300 patients sought to be included in the study.

### Statistical analysis

Data entry was verified by means of a 20% double data entry and a 100% double data check regarding the rate and time of patients until return-to-work. Participants who did and did not whish to participate were analysed on age using Student's t-test. All analyses were performed according to the intention-to-treat principle, which meant that all patients were included in the analysis. We censored patients who dropped out of the study because of missing data. Therefore, differences between patients who dropped out or completed the study were analysed according to their baseline quality of life scores.

All data were analysed by means of descriptive statics using PASW version 18. The baseline data were assessed to evaluate whether there was an imbalance between the intervention group and the control group using Student's t-test for continuous variables and the χ2 test for categorical variables. We considered a p-value≤0.05 to be statistically significant.

We calculated relative risks and 95% confidence interval for returning to work (full and partial) at 12 months of follow-up for the intervention group versus the control group. The median time until return-to-work was analysed with a Kaplan-Meier survival analysis, and differences between groups were tested with the log rank test. In addition, the Cox proportional hazard model of survival analysis was applied to estimate hazard ratios and the corresponding 95% confidence intervals for the time until return-to-work (full and partial) with a hazard ratio <1 indicating a longer time to return-to-work.

Improvements in the subsequent primary outcome of quality of life and the secondary outcomes of work ability and work functioning between groups were examined using a longitudinal multilevel analysis. Mean costs between the groups were analysed using Student's t-test.

## Results

**Figure 1:**
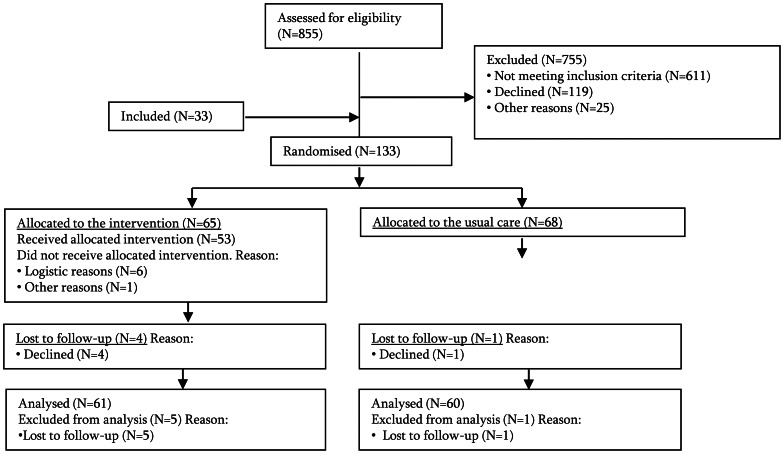
Patient flow.

Cancer patients who were diagnosed at one of the participating hospital departments between May 2009 and December 2010 and who were eligible and willing to participate were enrolled in the study. The enrolment of new patients ended in December 2010 to enable the inclusion of patient follow-up data within the time constraints of the study. A total of 755 of the 855 cancer patients were excluded; 611 did not meet the eligibility criteria primarily because they were too old, 119 declined participation, and 25 were excluded for other reasons, and this led to an overall response rate of 47% (Figure 1). In total, 133 cancer patients were included in the study; 65 were assigned to the intervention group and 68 were assigned to the control group. At baseline, all 133 patients provided complete data on the primary outcome, whereas 132 (99%) patients provided complete data on the secondary outcomes (Figure 1). The response rate at 12 months of follow-up was 128 (96%) for the outcome of return-to-work and was 108 (81%) for the outcome of quality of life and secondary outcomes. The reason why patients did not return the questionnaire included cancer recurrence (4 patients; 3%), decline (6;5%) or were unknown 11 (8%), while 4 (3%) patients died within the 12-months follow-up period (Figure 1). Patients were on average 47.5±7.9 years old. Breast cancer was the most common diagnosis (62%), followed by cancer diagnosis of the female reproductive system (34%) (Table 1). No statistically significant differences between the intervention group and the control group on any of the socio-demographic or prognostic characteristics measured at baseline or any medical characteristics measured at follow-up were identified (Table 1).

**Table 1:** Patient characteristics.

Patient characteristics		Intervention group *(N = 65)*	Control group *(N = 68)*	P-value **
**Socio-demographic characteristics** *				
Age *(years) * ¥		47.5±8.2	47.6±7.8	0.92
Gender (*% female)*		99%	100%	0.31
Marital status *(% married or living with partner)*		79%	69%	0.20
Breadwinner position *(% sole or shared)*		65%	56%	0.36
Education level (*%)*	Low	11%	16%	0.53
	Intermediate	59%	51%	
	High	30%	33%	
**Clinical characteristics** *				
Diagnosis	Breast cancer	64%	60%	0.82
(*%)*	Cervix cancer	23%	22%	
	Ovarian cancer	5%	10%	
	Vulva cancer	3%	3%	
	Other	5%	5%	
Number of co-	0	45%	54%	0.09
morbidities	1	22%	31%	
(*%)*	≥2	33%	15%	
Surgery *(%)*		99%	96%	0.78
Chemotherapy *(%)*		66%	71%	0.84
Radiotherapy *(%)*		60%	58%	0.67
**Work-related characteristics** *				
Type of occupation	Health care/education	38%	37%	0.69
(*%)*	Administrative	9%	9%	
	Sales	5%	12%	
	Other	48%	42%	
Type of work *(% mainly physically work)*		32%	40%	0.38
Physical workload *(0–28)* **		4.7±3.6	5.7±4.4	0.18
Time since sick listed *(days)*		26.5±35.1	15.0±53.2	0.15
Importance of work *(0–100)* **		58.7±23.1	51.5±28.3	0.11
Shift work *(% shift work)*		26%	19%	0.36
Type of contract	Permanent	89%	84%	0.17
*(%)*	Temporary	11%	9%	
	Self-employed	0%	4%	
	Other	0%	3%	
**Health-related characteristics** *				
Fatigue *(MFI* **	General fatigue *(0–20)*	12.4±4.9	13.1±4.3	0.37
Depression *(CES-D)* **	Sum score *(0–60)*	14.1±9.3	13.5±7.7	0.67
Self-efficacy *(ALCOS)* **	Sum score *(0–80)*	66.5±8.6	66.2±7.6	0.83

* Continuous variables: mean ± standard deviation; nominal and ordinal variables percentages.

** Higher scores represent higher level of physical workload, importance of work, fatigue, feelings of depression, and self-efficacy.

¥ Age at the time of randomisation.

### Hospital-based work support intervention

No harm or unintended effects were reported by patients as a result of participating in the intervention.

Seven patients (12%) assigned to the intervention group did not receive the patient education and support from the nurse [34]. For all patients who provided this type of consent (86%), at least one letter from the treating physician was sent to the occupational physician. In five cases (10%), the patients' occupational physician organized a meeting between the patient, supervisor, and himself to draw-up a return-to-work plan.

The median number of contacts made between the nurse and the patient was 4 (range 1–4) and the median duration of each meeting was 23 minutes (range 7–60). Eight (12%) patients assigned to the control group reported having received patient education or support regarding their return-to-work from their nurse.

### Primary outcome – return-to-work and quality of life

The return-to-work rate (full or partial) of all 128 randomised patients with follow-up data at 12 months was 79% for the intervention group and 79% for the control group (p = 0.97), and these rates were 86% and 83%, respectively (p = 0.61), when patients who died within the follow-up period or those with a life expectancy of only a few months were excluded. The relative risk of returning to work (full or partial) for the intervention group versus the control group was 1.03 (95% CI 0.84–1.2). Of the patients who did not return-to-work (intervention versus control group); 2 versus 2 died, 3 versus 1 had a life expectancy of few months, 4 versus 5 lost their jobs, 2 versus 5 experienced adverse side-effects, and 2 versus 0 had other reasons.

**Figure 2:**
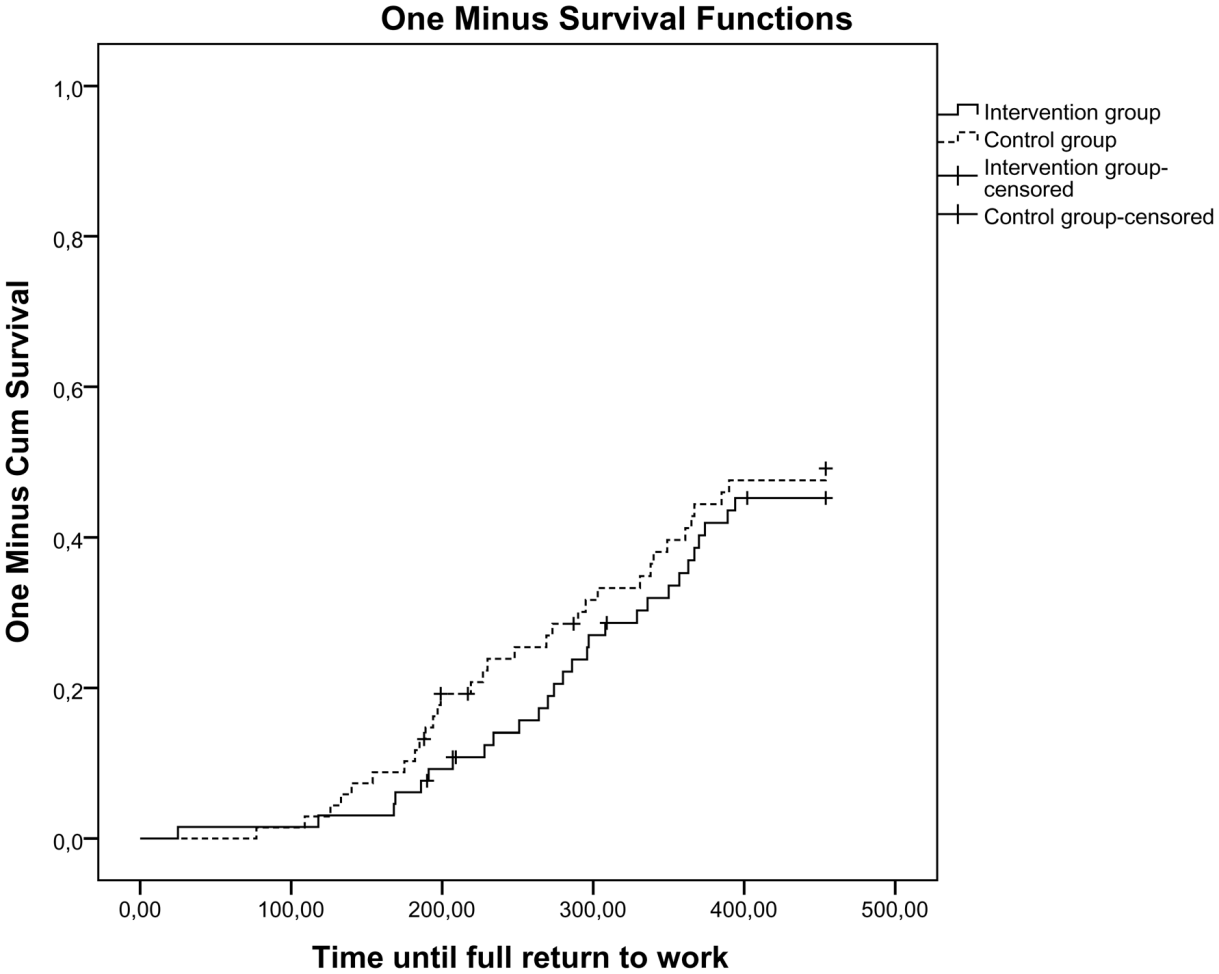
Kaplan-Meier survival curves for time to partial return-to-work (a) and full return-to-work (b).

Median time from the initial sick leave until partial return-to-work was 194 days (range 14–435) for the intervention group and 192 days (range 82–465) for the control group (log rank test; p = 0.90). Median time from initial sick leave until full return-to-work was 283 days (range 25–394) for the intervention group and 239 days (range 77–454) for the control group (log rank test; p = 0.52). Figure 2 summarizes Kaplan-Meier survival analyses for the two groups on partial and full return-to-work. The hazard ratio for partial return-to-work was 1.03 (95% CI 0.64–1.6) for the intervention group versus the control group and was 0.88 (95% CI 0.53–1.5) regarding full return-to-work.

Quality of life scores showed statistically significant improvements over time (p ranged between 0.014 to ≤0.001) but did not differ statistically significant between groups (p ranged between 0.15 to 0.99) (Table 2).

**Table 2:** Quality of life, work ability, and work functioning.

		Group	Baseline	6 months follow-up	12 months follow-up	P-value**
Quality of life* *(SF-*	Physical functioning	Intervention	76±28	71±21	81±16	0.95
*36) (0–100)*		Control	73±28	70±22	79±20	
*(N = 133)*	Role-physical	Intervention	48±44	29±40	47±40	0.46
		Control	50±43	31±37	61±41	
	Vitality	Intervention	60±21	51±20	59±19	0.60
		Control	57±17	51±16	56±16	
	General health	Intervention	61±21	54±18	64±17	0.15
		Control	61±18	59±18	70±19	
	Social functioning	Intervention	70±23	66±24	75±20	0.46
		Control	68±22	66±22	78±20	
	Role-emotional	Intervention	49±44	53±45	64±42	0.71
		Control	52±41	64±44	71±40	
	Mental health	Intervention	65±17	71±16	77±15	0.32
		Control	64±16	70±16	72±15	
	Pain	Intervention	69±30	67±25	75±21	0.99
		Control	70±23	69±20	76±17	
Quality of life*		Intervention	60±22	62±23	73±17	0.26
VAS *(0–100) (N = 133)*		Control	61±21	67±18	70±17	
Overall work ability*		Intervention	5±3	4±3	6±2	0.59
(WAI) *(0–10) (N = 133)*		Control	5±3	5±3	7±2	
Overall work productivity*		Intervention	NA	34±19	29±15	0.68
(WLQ) *(0–100) (N = 100)*		Control	NA	30±14	27±16	

Mean ± sd;

* Higher scores represent a higher level of functioning/well-being/quality of life, work ability, and work functioning.

** P-value represents the interaction effect of time and group.

### Secondary outcomes – work ability, work productivity, and costs

Work ability improved statistically significant over time (p≤0.001) but did not differ statistically significant between groups (p = 0.58) (Table 2). Work functioning did not improve significantly over time (p = 0.3) and did not differ significantly between groups (p = 0.48) (Table 2).

Table 3 shows that the intervention costs were 119 Euros per patient in the intervention group. The mean (± SD) lost productivity cost according to the human capital approach was 41.393 (±39.269) Euros in the intervention group and 38.968 (±38.399) Euros in the control group. The mean (± SD) lost productivity cost according to the friction costs approach was 14.030 (±3.614) Euros in the intervention group and 13.529 (±3.313) Euros in the control group. The mean work accommodations cost was 2.975 and 3.025 Euros in the intervention group and control group, respectively. These costs did not differ statistically between groups.

**Table 3:** Economic evaluation.

Costs of the work-directed intervention in Euros		
Description		Costs (€)
Training costs	1 trainer, time investment 24 hours, 50 Euros per hour	1200
	Study material, refreshments	125
	Attendance costs nurses, 11 nurses, 30 Euros per hours, 4 hours	1320
Total training costs per patient in the intervention group	41	
Work-directed intervention	Mean hour of investment of nurse was 1.2 hour, 43 Euros per hour	66
	Mean hour of investment of secretary was 0.16 hour, 30 Euros per hour	5
	Informational leaflet	7
Total intervention costs per patient in the intervention group	78	
Total costs per patient in the intervention group	119	

Costs differences between groups in Euros					
	N	Intervention group	Control group	Mean difference	P-value
Productivity loss net HCA *Mean (±SD) Euros*	128	41393 (±39269)	38968 (±38399)	−2425	0.72
Productivity loss net FCA	128	14030 (±3614)	13529 (±3313)	−438	0.48
*Mean (±SD) Euros*					
Work adjustments	3*	2975	3025 (±71)	50	0.67
*Mean (±SD) Euros*					
Work-directed intervention *Mean Euros*	128	119	0	−119	NA

Abbreviations: HCA Human Capital Approach; FCA Friction Costs Approach; NA: not applicable; SD = standard deviation.

* Only three patients had work adjustments that were not related to productivity.

## Discussion

The objective of this study was to determine the effect of a hospital-based work support intervention for female cancer patients on return-to-work and quality of life. In general, return-to-work rates were high. We failed to show any differences between groups on return-to-work outcomes and quality of life scores.

### Strengths and limitations

One strength of our study was the innovative approach that was used to address the adverse work outcomes of cancer patients. Few studies have addressed this important subject by developing an intervention that is primarily aimed at enhancing the return-to-work of cancer patients [12], [13], [34]. Furthermore, another strength of this study was the use of a low-cost intervention that could be implemented without substantially increasing the time required, which is important because of the burden on cancer care. In addition, this intervention was easily adapted to the existing variation in usual psycho-oncological care, which yields high external validity. One limitation of our study was the inability to include sufficient patients, according to our predetermined power analysis, which led to greater uncertainty in the results.

### Interpretation of findings

We found that the intervention was easily accepted in usual psycho-oncological care and we found that patients were notably satisfied with the intervention [34]. For those reasons, addressing the return to work of cancer patients is highly relevant for usual psycho-oncological care. We found similar return-to-work outcomes and quality of life scores for both groups. There are several possible explanations for the lack of statistically significant difference between groups, which can be sought in the intervention content and the study design. The basic assumption behind the intervention was that return to work would increase by means of improved self-assessed work ability as a result of patient education and support that addressed misconceptions about cancer and work. We found that self-assessed work ability increased significantly over time but did not differ significantly between groups. It is possible that addressing these misconceptions could have required a more intense intervention or that the training we provided to the nurses was not sufficient. We do not know precisely which misconceptions impede return to work and which should be addressed. On the other hand, this later possibility was indicated as a number of nurses mentioned that they were not completely convinced of their competence to deliver the return-to-work advice. It may be that our half-day training course was too short to enable nurses to gain the knowledge required to adequately address patients' misconceptions about return to work adequately. For these reasons, it is possible that certain misconceptions regarding cancer and work could have persisted and may have resulted in the absence of an intervention effect.

In addition, we experienced difficulties in involving the occupational physician and the employer for the intervention while their involvement appeared to be important [35] and may have caused the absence of an intervention effect.

There are some observational studies that showed that the treating physician's advice about return-to-work influenced work resumption considerably either with a shorter or with a longer return-to-work [16], [17], [34]. However, our study shows that apparently this is an overestimation that is not reproduced in an experimental study.

### Methodological considerations

Another potential explanation for similar findings between groups may be related to the study design. Several sources of potential bias may have influenced our findings. To start with, the contrast between groups may have been reduced in several ways. The quality of usual care regarding work advice was probably higher in hospital departments that were willing and able to participate at the onset of the study compared to those that were not willing or able to participate, as nurses who worked in hospital departments that participated recognised the importance of work for cancer patients prior to the study. Furthermore, we attempted to reduce contamination between groups by separating the nurses who delivered the intervention from those who delivered usual care. However, this separation was not possible in all cases, and therefore contamination occurred to a larger extent. Next, the contrast between groups may have been reduced due to the fact that all cancer patients were informed about the general aim of the study (i.e. information bias). Finally, the contrast between groups may have been reduced due to a patient selection bias; patients participating in this study may already be of the opinion that work is an important subject that should receive attention.

In accordance with the intention to treat analysis we included in the survival analysis patients who died within the follow-up period as censored. However, an assumption in survival analysis is that when a patient is censored, the change that a patient will be able to achieve the outcome is still 50% [36], [37], which is not the case in this situation. However, on a population of 133 patients, we do not expect that the 4 patients who were equally divided between the intervention group and control group, influenced the findings significantly.

### External validity

It is generally acknowledged that the disability legalisation of a country influences the return-to-work outcomes of employees on sick leave and that disability legalisations varies widely among countries [38]. For that reason, the effect of interventions on return-to-work may also vary from one country to another. The results and conclusions of this study are relevant for the Netherlands due to its social security legislation. However, the early hospital-based work support intervention integrated into usual psycho-oncological care could be adapted and generalised to other countries because cancer patients in other countries experiencing a lack of support about their return-to-work from the hospital as often as patients in the Netherlands [39]. The exact content of the intervention should be adapted to the social security legislation of the country it is implemented in.

### Recommendations for further research and practice

In terms of recommendations for clinical practice, this study revealed that psycho-oncological care can address the return-to-work of cancer patients early in their treatment, as well as follow-up, as the intervention was appreciated by patients and was perceived as useful and feasible by the nurses. Since, the occupational physician and employer involvement is pivotal for a successful return to work but appeared problematic in our intervention, it seems important to solve what impedes their involvement and to adapt the intervention accordingly. As we found similar work outcomes between the intervention group and the control group, a recommendation for further research is to study if an improved intervention leads to shorter time to return-to-work.

Due to the large range in time to return to work, it seems important to identify patients who have a higher risk of getting adverse work outcomes based on a clinical prediction rule. Therefore, a recommendation for further research is, to develop such a clinical prediction rule for work outcomes and to evaluate it for the accuracy in identifying patients with a higher risk of adverse work outcomes. Furthermore, apart from identifying patients with a higher risk, it is also important to make the intervention more tailored as it appeared that some patients do not need an intervention to achieve a successful return to work while other patients might have benefited from a more intense intervention. A possibility to make an intervention more tailored is by using a stepped care model. This means that a low-intensity intervention can be offered to all patients while a high-intensity intervention is only offered to patients, for whom work resumption turns out to be problematic.

We found that the contrast between groups was reduced, due to the study design. Therefore, another recommendation for further research would be to consider alternative study designs, such as a cluster randomised controlled trial [40].

## Supporting Information

Checklist S1 **CONSORT Checklist.**
(DOC)

Protocol S1 **Trial Protocol.**
(PDF)
