# Supplementary material for: Effectiveness of a Hospital-Based Work Support Intervention for Female Cancer Patients – A Multi-Centre Randomised Controlled Trial
Source: PLoS One. 2013 May 22;8(5):e63271. doi: 10.1371/journal.pone.0063271 (PMC3661555; doi:10.1371/journal.pone.0063271)
Supplement: Protocol S1 — Trial Protocol. (PDF) [file pone.0063271.s002.pdf]

RESEARCH PROTOCOL

PROTOCOL TITLE 'To enhance return-to-work in cancer patients – a randomised controlled trial '

Protocol ID	NL24840.018.08
Short title	To enhance return-to-work in cancer patients
Version	4
Date	17-juli-2009
Coordinating investigator/project leader	Dr. A.G.E.M de Boer<br>Meibergdreef 9<br>1105 AZ Amsterdam<br>020-5665323<br>a.g.deboer@amc.uva.nl
Principal investigator(s) (in Dutch: hoofdonderzoeker/uitvoerder)	Dr. A.G.E.M de Boer<br>Meibergdreef 9<br>1105 AZ Amsterdam<br>020-5665323<br>a.g.deboer@amc.uva.nl
Sponsor (in Dutch: verrichter/opdrachtgever)	Stichting Instituut GAK (SIG)
Independent physician(s)	Dr.H. Wind<br>Meibergdreef 9<br>1105 AZ Amsterdam<br>020-5663878<br>h.wind@amc.uva.nl
Laboratory sites	Not applicable
Pharmacy	Not applicable

PROTOCOL SIGNATURE SHEET

Name	Signature	Date
Sponsor or legal representative:<br><br>For non-commercial research,<br>Head of Department:<br>Prof. Dr. M.H.W. Frings-Dresen	*Not applicable*	*Not applicable*
Coordinating Investigator/Project leader/Principal Investigator:<br>Dr. A.G.E.M. de Boer		

TABLE OF CONTENTS

1. INTRODUCTION AND RATIONALE 8
2. OBJECTIVES 9
3. STUDY DESIGN 10
4. STUDY POPULATION 11
4.1 Population (base) 11
4.2 Inclusion criteria 11
4.3 Exclusion criteria 11
4.4 Sample size calculation 11
5. TREATMENT OF SUBJECTS 12
5.1 Investigational product/treatment 12
5.2 Use of co-intervention 13
5.3 Escape medication 13
6. INVESTIGATIONAL MEDICINAL PRODUCT 14
7. METHODS 14
7.1 Study parameters/endpoints 14
7.1.1 Main study parameter/endpoint 14
7.1.2 Secondary study parameters/endpoints 14
7.1.3 Other study parameters 14
7.2 Randomisation, blinding and treatment allocation 15
7.3 Study procedures 15
7.4 Withdrawal of individual subjects 17
7.4.1 Specific criteria for withdrawal 17
7.5 Replacement of individual subjects after withdrawal 17
7.6 Follow-up of subjects withdrawn from treatment 17
7.7 Premature termination of the study 17
8. SAFETY REPORTING 17
8.1 Section 10 WMO event 17
8.2 Adverse and serious adverse events 17
8.2.1 Suspected unexpected serious adverse reactions (SUSAR) 17
8.2.2 Annual safety report 18
8.3 Follow-up of adverse events 18
8.4 Data Safety Monitoring Board (DSMB) 18
9. STATISTICAL ANALYSIS 19
9.1 Descriptive statistics 19
9.2 Univariate analysis 19
9.3 Multivariate analysis 19
9.4 Interim analysis 19
10. ETHICAL CONSIDERATIONS 19
10.1 Regulation statement 19
10.2 Recruitment and consent 19
10.3 Objection by minors or incapacitated subjects 19

10.4 Benefits and risks assessment, group relatedness 20
10.5 Compensation for injury 20
10.6 Incentives 20
11. ADMINISTRATIVE ASPECTS AND PUBLICATION 20
11.1 Handling and storage of data and documents 20
11.2 Amendments 20
11.3 Annual progress report 20
11.4 End of study report 21
12. REFERENCES 22

LIST OF ABBREVIATIONS AND RELEVANT DEFINITIONS

ABR	ABR form (General Assessment and Registration form) is the application form that is required for submission to the accredited Ethics Committee (ABR = Algemene Beoordeling en Registratie)
AE	Adverse Event
AR	Adverse Reaction
CA	Competent Authority
CCMO	Central Committee on Research Involving Human Subjects
CV	Curriculum Vitae
DSMB	Data Safety Monitoring Board
EU	European Union
EudraCT	European drug regulatory affairs Clinical Trials GCP Good Clinical Practice
IB	Investigator’s Brochure
IC	Informed Consent
IMP	Investigational Medicinal Product
IMPD	Investigational Medicinal Product Dossier
METC	Medical research ethics committee (MREC); in Dutch: medisch ethische toetsing commissie (METC)
(S)AE	Serious Adverse Event
SPC	Summary of Product Characteristics (in Dutch: officiële productinfomatie IB1-tekst)
Sponsor	The sponsor is the party that commissions the organisation or performance of the research, for example a pharmaceutical company, academic hospital, scientific organisation or investigator. A party that provides funding for a study but does not commission it is not regarded as the sponsor, but referred to as a subsidising party.
SUSAR	Suspected Unexpected Serious Adverse Reaction
Wbp	Personal Data Protection Act (in Dutch: Wet Bescherming Persoonsgevens)
WMO	Medical Research Involving Human Subjects Act (Wet Medisch-wetenschappelijk Onderzoek met Mensen)

SUMMARY

Rationale: Survival rates of cancer have been increasing in recent years. It is generally assumed that the incidence of cancer in the working population in western countries will increase as well (Morell and Pryce 2005). For many cancer patients, cancer has become a chronic disease which causes poorer general health outcomes in comparison to the general population (Yabroff et al. 2004). The burden of the diseases itself and the treatment affects quality of life in all its aspects and one of these aspects is return-to-work (Main et al. 2005). Earlier research showed that not all cancer patients who were working prior to their diagnosis, returned to work (Spelten et al. 2002). Moreover, cancer patients have the highest prevalence of work impairments in comparison to patients with other chronic illnesses (Kessler et al. 2001). To reduce these negative consequences for cancer patients as well as for the society at large an intervention has been developed to enhance return-to-work. The intervention will be carried out by a nurse who will provide counselling according to a special developed protocol. The hypothesis is that the patients who were counselled according to the intervention will return-to-work earlier and will have a better quality of life than patients who were counselled according to usual care.

Objective: <u>Primary objective</u>: to determine the effect of the intervention on return-to-work and quality of life. <u>Secondary objectives:</u> To determine the effect of the intervention on the work ability and on the work limitations. To determine the feasibility of the intervention and to determine the direct and indirect costs of the intervention.

Study design: Randomised controlled trial with a follow-up of 24 months. Patients will be randomised to a control group or to an intervention group. Patients in the control group will get care as usual and patients in the intervention group will get the intervention.

Study population: Patients with a primary diagnosis of cancer, 18 - 60 years old.

Intervention: A vocational rehabilitation intervention. Patients in the control group will be counselled according to usual care and patients in the intervention group will be counselled according to a special developed protocol (the intervention). The intervention will consist of 4 appointments with the nurse at the hospital (total 1.00 hour) and of 1 meeting of half an hour with patient’s manager and patient’s occupational physician.

Main study parameters/endpoints: Return-to-work and quality of life.

Nature and extent of the burden and risks associated with participation, benefit and group relatedness: The burden for the patients in the control group will be 3.00 hours for filling out 6 questionnaires. The burden for the patients in the intervention group will be 1.50 hours to completed the intervention plus 3.00 hours for filling out 6 questionnaires. There are no risks associated with participation.

1. INTRODUCTION AND RATIONALE

Survival rates of cancer have been increasing in recent years as a result of screening, earlier and better diagnosis and advanced treatment. It is generally assumed that the incidence of cancer in the working population in western countries will increase due to ageing of the working population, due to the fact that people have to work longer until their retirement and due to the improved cure of other diseases (Morell and Pryce 2005). The consequence of this increasing incidence and increasing survival rates will be that cancer patients will become more common in the workplace and should be a significant concern for the employer (Morell and Pryce 2005).

Although treatment has improved in the recent years, cancer patients are still suffering from the effects of diagnosis and treatment for cancer resulting in long-term physical complaints, depression, fatigue and distress (Carlson et al. 2004; Reich et al. 2008). For many cancer patients, cancer has become a chronic disease which causes poorer general health outcomes in comparison to the general population (Yabroff et al. 2004). The burden of the diseases itself and the treatment affects quality of life in all its aspects and one of these aspects is the preservation of work or return-to-work (Main et al. 2005). The inability to work caused by the residual long-term symptoms may result in a lower quality of life, lower self-esteem, social isolation and financial loss (Ferrell et al. 1992a,b; Lauzier et al. 2008). In contrast, working gives a sense of normality, distraction, and is seen as an important part of recovery (Clark and Landis 1989; Peteet 2000). Not being able to work is not only a loss for cancer patients, but also for the society at large due to absenteeism and lost productivity (Verbeek and Spelten 2007).

Earlier research showed that not all cancer patients who were working prior to their diagnosis, returned to work (Spelten et al. 2003). Furthermore, cancer patients who did (partly) return-to-work still had a lower work ability and/or suffered from loss of productivity in comparison to the general population (Kessler et al. 2001; Feuerstein et al. 2007). Moreover, cancer patients have the highest prevalence of work impairments in comparison to patients with other chronic illnesses (Kessler et al. 2001; Short et al. 2008). Cancer patients also experience problems regarding job discrimination, hostility in the workplace and lack of emotional and practical support from managers and/or from occupational health services (Maunsell et al. 2004; Morrell and Pryce et al. 2005; Taskila et al. 2006; Verbeek and Spelten 2007).

A vocational rehabilitation program special developed for cancer patients may reduce these negative consequences for cancer patients as for the society at large. Our systematic literature review about interventions aimed at return-to-work in cancer patients revealed that 3 studies were primarily aimed at return-to-work in cancer patients. None of these interventions were evaluated in a randomised controlled trial. It was therefore impossible to

determine which intervention (or part of it) was most effective on return-to-work. Criteria were made to determine which intervention was for instance most feasible to carry out. On the basis of these findings, a pilot study about this topic which was carried out in the AMC (Nieuwenhuijsen et al. 2006), experiences from earlier research about cancer and work (De Boer et al. 2008; Nieuwenhuijsen et al. 2006; Spelten et al. 2002, 2003; Taskila et al. 2006, 2007a,b; Verbeek et al. 2003), interviews with oncologic nurses and a radiotherapist (unpublished data) and on return-to-work interventions in other chronic illness, the intervention was developed. The intervention will be, as good as possible, fit in the normal cancer care, in that way the burden for the patients will be as less as possible and the burden for the departments as well. Therefore, nurses who are already integrated in normal cancer care will carry out the intervention. In normal cancer care consultation hours are scheduled between the nurse and the patient to provide psycho-oncologic care. Work is not a general issue during these meetings. The meetings within the context of the intervention will be scheduled as part of these consultation hours. If the study points out that the intervention is effective, the intention is to implement the intervention in normal cancer care. This study is supported by the Dutch federation of cancer patients associations (NFK).

2. OBJECTIVES

Primary objective: To determine the effect of the intervention on return-to-work and quality of life.

Secondary objectives: To determine the effect of the intervention on the work ability and on the work limitations. To determine the feasibility of the intervention and the direct and indirect costs of the intervention.

3. STUDY DESIGN

The study will be carried out as a randomised controlled trial with a follow-up of 24 months (figure 1). Eligible patients who signed informed consent will be randomised to the control group and will get care as usual or to the intervention group and will get the intervention. The the intervention will be carried out by a nurse (or by another professional who provides psycho-oncology care in the usual care). Before the start of the inclusion of patients, at least 2 nurses per department will be randomised to carry out care as usual or the intervention. Resulting per department in at least 1 nurse who will provide counselling according to the intervention and in 1 nurse who will provide care as usual.

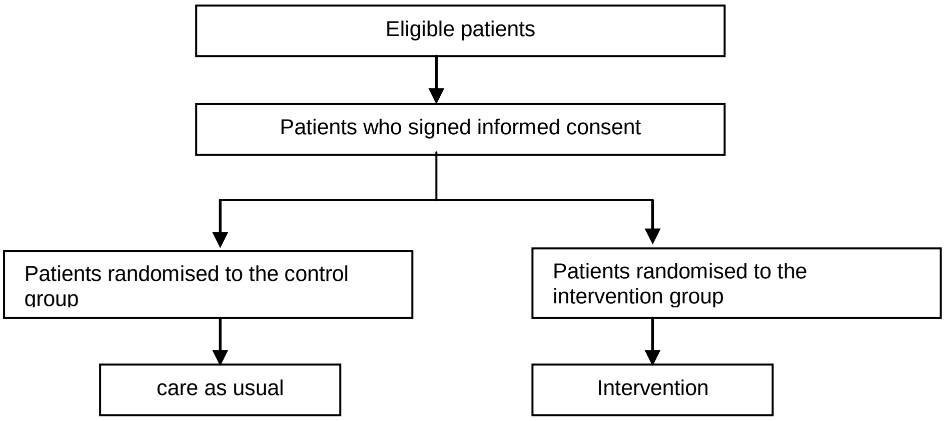

Figure 1: Study design

4. STUDY POPULATION

4.1 Population (base)

Patients primary diagnosed with cancer treated in the department of gynaecology or surgery of the AMC, of the department of surgery of the RdGG and of the department of surgery and gynaecology of the ASz. Except for patients diagnosed with a primary diagnosis of testis cancer, non-melanoma or melanoma skin cancer since it is known that these patients do not experience significant problems with return-to-work (Spelten et al. 2003; Taskila-Åbrandt et al. 2005). Therefore, the intervention would not be of use for this patient group.

4.2 Inclusion criteria

- Primary diagnosis of cancer with an one year survival rate of approximately 80% and treatment with curative intent
- Age between 18 and 60 years
- Paid employment at the time of diagnosis
- Sick listed

4.3 Exclusion criteria

- Not able to understand, speak, read or write Dutch sufficiently
- Severe mental disorder or other severe co-morbidity
- Primary diagnosis of cancer has been made more than two months ago
- Patients who visit the hospital for a second opinion
- Primary diagnosis of testis cancer
- Primary diagnosis of non-melanoma or melanoma skin cancer

4.4 Sample size calculation

The calculation of the sample size is based on two earlier studies on return-to-work in cancer patients. Based on the study of Spelten et al. (2003) with consecutive cancer patients, the expectation will be that 18 months after the diagnosis 64% of the patients have returned to work (Spelten et al. 2003). Based on the study of Nieuwenhuijsen et al. (2006) on the effect of providing patient education regarding return-to-work and enhancing provider communication, the expectation will be that 81% of the patients have returned to work 18 months after diagnosis (Nieuwenhuijsen et al. 2006). Due to the in- and exclusion criteria, a part of the patients with not much return-to-work problems will not be included in this study, which may lead to less favourable return-to-work rates. However, the intervention is more extended than in the study of Nieuwenhuijsen et al. (2006). Therefore, the expectation will be that the return-to-work rates will be the same as in the study of Nieuwenhuijsen et al. (2006),

resulting in a Odds Ratio (OR) of 2.4 of the intervention versus care as usual. With a power of 80% and two-sided significant level of $p<0.05$, the sample size calculation indicates that the sample size should be 108 patients in every arm and therefore a total of 216 patients. Assuming that 20% of the initial patients will be lost to follow-up during the study, 270 patients have to be recruited to be able to gather data for analysis at 18 months on 216 patients. To account for at least 10% missing data at baseline, 300 patients will be included.

5. TREATMENT OF SUBJECTS

5.1 Investigational product/treatment

The intervention will be a vocational rehabilitation program which consist of 4 meetings between the patient and the nurse at the hospital as a component of the normal consulting hour (total 1 hour extra) and of 1 meeting with the patient's manager and patient's occupational physician (0.5 hour). If a patient is returned to work or if a patient does not have consulting hours at the hospital anymore, the meetings will be replaced by contact by telephone. The intervention will be discussed in detail below.

T_1: During the first meeting, which will take place a couple of days after baseline, the nurse will give general information about return-to-work, social security, rights/obligations according to the Improved Gatekeepers Act and the educational leaflet of 10-steps of advice will be given as well to the patients. The nurse will take a short work-anamnesis and will guide the patients with the decision to work during and/or after treatment and will discuss the meaning of work, as well as about the best ways (personal style and comfort) to inform their colleagues and manager about the diagnosis and treatment and the preferences of the patients to keep their colleagues and manager informed during treatment/aftercare. At the end of the first meeting, the appointment for the second meeting will be made and depends on diagnosis, treatment and preference of the patients. If patient gives consent that the treating physician provides medical information to the occupational physician, general medical information from the treating physician as well as general information about the study (including the educational leaflet) will be send to patients' occupational physician (diagnosis, prognosis and treatment plan) after this first meeting.

T_2: The second meeting will be scheduled at a maximum of 10 months after the first meeting, however the aim is that it will be scheduled after the end of the medical treatment. During this meeting the subjects of the first meeting will be discussed such as communication with colleagues, manager and occupational physician. In collaboration with the patient, ideas about return-to-work/work adjustments on the basis of the work-

anamnesis and physical and psychosocial limitations/problems will be made. The consequences of return-to-work or staying on sick leave will be taken into account. After this second meeting, general medical information about the outcome of the treatment will be send to the occupational physician as well as the advice from the nurse concerning return-to-work and work adjustments (if patient gave consent). The occupational physician will be advised to schedule a meeting with the patient and patient's manager to put these advice into action by making a return-to-work plan. The idea of the return-to-work plan is that it contains exact data and task(s): for instance first day of return-to-work, number of hours the patient will work, the task(s) he/she is going to do and with which steps patient will increase working hours, days of working and/or will do more/different tasks. The patient will be asked to send a copy of the return-to-work plan to the nurse.

T_3: During the third meeting, which will take place at a maximum of 2 months after the second meeting, return-to-work, sick leave and return-to-work plan will be discussed. Stagnation and success of the components of the return-to-work plan will be discussed as well as actions to solve these problems will be provided. If necessary the return-to-work plan will be altered or extra information or advice will be provided. Also, possible medical or psychosocial problems will be discussed and if necessary the patient will be referred.

T_4: There are two options for the fourth meeting: patient is returned to work or patient is not returned to work. If a patient is returned to work, contact by telephone will be carried out by the nurse in which advice to stay at work will be provided as well as extra information will be given or work adjustments will be discussed if necessary. If patient is not returned to work, the fourth meeting will take place at a maximum of 14 months after baseline, the subjects of the third meeting will be discussed.

5.2 Use of co-intervention

Subjects will be able to use any co-intervention they want to do. Since, it is likely that other vocational rehabilitation programs will have a significant effect on return-to-work, these will be monitored by asking the patients at the end of the intervention if they had participated in any other vocational rehabilitation program(s). In that way we will be able to adjust for if necessary. Since it is unknown what the effect of other kinds of rehabilitation programs (such as an exercise program) is on return-to-work these will not be assessed.

5.3 Escape medication

Not applicable

6. INVESTIGATIONAL MEDICINAL PRODUCT

Not applicable

7. METHODS

7.1 Study parameters/endpoints

7.1.1 Main study parameter/endpoint

The primary outcome parameters are return-to-work and quality of life.

Return-to-work is defined as time to partial or full return-to-work, meaning number of calendar days between first day of sick leave and first day at work. The patient must work (part time or full time) for at least 4 weeks successively.

7.1.2 Secondary study parameters/endpoints

The secondary outcome parameters are:

- Work ability
- Work limitations
- Feasibility
  - Feasibility of the procedure
  - Satisfaction with the intervention (nurses who will carry out the intervention, patients in the intervention and control group, treating physician, occupational physician and patients' manager)
  - Protocol adherence of the nurse who will carry out the intervention
  - Process evaluation of the usual care, carried out by the nurses who carry out care as usual
  - Compliance of the patients, in the intervention group, with the intervention
- Direct/indirect costs of the intervention
  - Absenteeism
  - Work productivity
  - Work adjustments
  - Costs to carry out the intervention

7.1.3 Other study parameters

The prognostic parameters are: age, gender, education, diagnosis, cancer treatment, number of working hours according to the contract, physical workload, importance of work, fatigue, depression, co-morbidity and self-efficacy.

The descriptive parameters are: first day of sick leave, marital status, ethnicity, time since date of diagnosis, breadwinner, position, shift work, years of present position, years of paid employment, income and size of company.

7.2 Randomisation, blinding and treatment allocation

Patients will be randomised to the control group and will get care as usual or to the intervention group and will get the intervention. Before the start of the inclusion of patients, at least 2 nurses per department will be randomised to carry out care as usual or the intervention. Resulting, per department in at least 1 nurse who will provide counselling according to the intervention and in 1 nurse who will provide counselling according to usual care.

The patients, nurses, the treating physicians and the researchers are not blinded for the group assignment. It is unlikely that patients of the same company participate in the study and therefore, it is unlikely that patients in the control group will get detailed information about the content of the intervention. Since all patient questionnaires will be filled out at home, no direct influence by the researcher is likely to occur.

7.3 Study procedures

All study parameters will be assessed with a questionnaires or checklists, except for the study parameter diagnosis and treatment which will be assessed on the basis of the medical files of the patients. All patient questionnaires can be filled out at home.

Patient

Patients in the control group and intervention group:

- Baseline questionnaire which will assess the primary, prognostic and descriptive parameters and the work ability (secondary parameter). This questionnaire contains the SF36 (quality of life), VAS (quality of life), VAS (importance of work), WAI (work ability), MFI (fatigue), CES-D (depression), ALCOS (self-efficacy), age, gender, marital status, ethnicity, education, diagnosis, time since date of diagnosis, cancer treatment, co-morbidity, first day of sick leave, breadwinner (sole or shared), number of working hours according to the contract, physical workload, shift work, years of present position, years of paid employment and income.
- The second, third, fourth and fifth patient questionnaire will be assessed respectively, 6, 12, 18 and 24 months after baseline. These questionnaires contain the primary, a part of the secondary outcome parameters and the prognostic parameters which change due to treatment: cancer treatment, the SF36 (quality of life), VAS (quality of life), VAS (importance of work), return-to-work, WAI (work ability), WL-27 (work

limitations), MFI (fatigue), CES-D (depression), ALCOS (self-efficacy), work adjustments and income

Patients in the intervention group:

- After the end of the intervention, a questionnaire about their satisfaction with and compliance to the intervention will be assessed.

Patients in the control group:

- After the last visit at the hospital, a questionnaire about their satisfaction with care as usual will be assessed.

Nurse

Nurses who carry out the intervention:

- The protocol adherence will be assessed by the researchers with a checklist on the basis of the reports for each patient at the end of the intervention.
- After the end of the study, the satisfaction with the intervention will be assessed by means of a questionnaire.

Nurses who carry out care as usual:

- Counselling in the usual care will be monitored with questionnaires, which will be filled out each two months about each patient counselled in the last two months.

Manager

Patients' manager of patients in the intervention group:

- If the meeting with the patient, patient's manager and patient's occupational physician has occurred and if a patient agrees that the researcher will contact his/her manager than the satisfaction with the intervention will be assessed by the researcher for each patient.

Treating physician

Treating physicians of patients in the control- and intervention group:

- After the end of the study, the satisfaction with the intervention will be assessed by questionnaire.

Researcher

- The feasibility of the procedure of the study as well as the costs to carry out the intervention will be assessed by the researchers at the end of the study with a checklist.

- The number of meetings with nurse, occupational physician and/or manager will be assessed by the researchers as well as the communication between the treating physician and occupational physician.

7.4 Withdrawal of individual subjects

Subjects can leave the study at any time for any reason if they wish to do so without any consequence affecting their normal cancer care. The reason for withdrawal will be required.

7.4.1 Specific criteria for withdrawal

Not applicable

7.5 Replacement of individual subjects after withdrawal

Not applicable

7.6 Follow-up of subjects withdrawn from treatment

Not applicable

7.7 Premature termination of the study

Not applicable

8. SAFETY REPORTING

8.1 Section 10 WMO event

In accordance to section 10, subsection 1, of the WMO, the investigator will inform the subjects and the reviewing accredited METC if anything occurs, on the basis of which it appears that the disadvantages of participation may be significantly greater than was foreseen in the research proposal. The study will be suspended pending further review by the accredited METC, except insofar as suspension would jeopardise the subjects' health. The investigator will take care that all subjects are kept informed.

8.2 Adverse and serious adverse events

Not applicable

8.2.1 Suspected unexpected serious adverse reactions (SUSAR)

Not applicable

8.2.2 Annual safety report

Not applicable

8.3 Follow-up of adverse events

All adverse events will be followed until they have abated, or until a stable situation has been reached. Depending on the event, follow up may require additional tests or medical procedures as indicated, and/or referral to the general physician or a medical specialist.

8.4 Data Safety Monitoring Board (DSMB)

Not applicable

9. STATISTICAL ANALYSIS

9.1 Descriptive statistics

All analyses, performed to distinguish differences between control group and intervention group will be performed according to the intention-to-treat principle. All data will be presented qualitative. All baseline data and all data of primary and secondary parameters will be presented using descriptive statistics.

9.2 Univariate analysis

9.3 Multivariate analysis

If necessary, the differences between control group and intervention group at baseline will be adjusted for with a Cox regression analyses as for confounders such as diagnosis. The number of days until return-to-work will be analysed with the Kaplan-Meier survival method and differences between groups will be tested with a log rank test. Longitudinal multivariate analysis will be used to examine differences in improvement of quality of life, work ability and of work limitations between control- and intervention group.

9.4 Interim analysis

Not applicable

10. ETHICAL CONSIDERATIONS

10.1 Regulation statement

This study will be conducted according to the principles of the Declaration of Helsinki (5th version, 2004) and in accordance with the Medical Research Involving Human Subjects Act (WMO).

10.2 Recruitment and consent

The treating physician and nurse will inform the patients about the study when the patient visit the treating physician to discuss their treatment plan. The treating physician will check the inclusion and exclusion criteria. After a week when patients visit the hospital (according to usual care), the researcher will ask for informed consent and if a patient signed informed consent the patient will be randomised to the control or intervention group.

10.3 Objection by minors or incapacitated subjects

Not applicable

10.4 Benefits and risks assessment, group relatedness

There are no risk associated with participating. The burden for the patients in the intervention group will be in proportion to the possible benefit of a higher change of return-to-work which is associated with less financial problems, higher self-esteem and higher quality of life. The burden for patients in the control group will be small.

10.5 Compensation for injury

The METC grants exemption from insurance, because there are no health risks of participation in this study.

10.6 Incentives

Not applicable

11. ADMINISTRATIVE ASPECTS AND PUBLICATION

11.1 Handling and storage of data and documents

Patients, patients' manager and nurses are registered by a code number. Code number and personal particulars are kept in an encrypted file, only accessible for the researchers. The data are kept for 15 years.

11.2 Amendments

Amendments are changes made to the research after a favourable opinion by the accredited METC has been given. All amendments will be notified to the METC that gave a favourable opinion.

All substantial amendments will be notified to the METC and to the competent authority.

11.3 Annual progress report

The sponsor/investigator will submit a summary of the progress of the trial to the accredited METC once a year. Information will be provided on the date of inclusion of the first subject, numbers of subjects included and numbers of subjects that have completed the trial, serious adverse events/ serious adverse reactions, other problems, and amendments.

11.4 End of study report

The investigator will notify the accredited METC of the end of the study within a period of 8 weeks. The end of the study is defined as patient's last visit.

In case the study is ended prematurely, the investigator will notify the accredited METC, including the reasons for the premature termination.

Within one and a half year after the end of the study, the investigator/sponsor will submit a final study report with the results of the study, including any publications/abstracts of the study, to the accredited METC. The reason for the extended time in which the final study report will be available is the fact that the follow-up is 24 months after baseline and 12 months after the last patient's visit.

Public disclosure and publication policy
The sponsor has put no limitations of any kind to publication of the results.

12. REFERENCES

Carlson, L. E., et al., "High levels of untreated distress and fatigue in cancer patients," Br. J. Cancer 90, 2297 (2004)

Clark, J. C. & Landis, L. L., "Reintegration and maintenance of employees with breast cancer in the workplace," AAOHN. J. 37, 186 (1989)

De Boer, A. G., et al., "Work ability and return-to-work in cancer patients," Br. J. Cancer 98, 1342 (2008)

Eldridge, S.M. et al. “informed patient consent to participation in cluster randomized trials: an empirical exploration of trials in primary care”. Clinical Trials. 2,91-98 (2005)

Ferrell, B., et al., "The meaning of quality of life for bone marrow transplant survivors. Part 1. The impact of bone marrow transplant on quality of life," Cancer Nurs. 15, 153 (1992a)

Ferrell, B., et al., "The meaning of quality of life for bone marrow transplant survivors. Part 2. Improving quality of life for bone marrow transplant survivors," Cancer Nurs. 15, 247 (1992b)

Feuerstein, M., et al., "Work productivity in brain tumor survivors," J. Occup. Environ. Med. 49, 803 (2007)

Kessler, R.C., et al. "the effects of chronic medical conditions on work loss and work cutback,” J Occup Environ Med. 43,218 (2001)

Lauzier, S., et al., "Wage losses in the year after breast cancer: extent and determinants among Canadian women," J. Natl. Cancer Inst. 100, 321 (2008)

Main, D. S., et al., "A qualitative study of work and work return in cancer survivors," Psychooncology. 14, 992 (2005)

Maunsell, E., et al., "Work situation after breast cancer: results from a population-based study," J. Natl. Cancer Inst. 96, 1813 (2004)

J. Morrell and J. Pryce, “How cancer affects working lives” Report No. 1-904370-93-4, 2005.

Nieuwenhuijsen, K., et al., "Enhanced provider communication and patient education regarding return to work in cancer survivors following curative treatment: a pilot study," J. Occup. Rehabil. 16, 647 (2006)

Peteet, J. R., "Cancer and the meaning of work," General Hospital Psychiatry. 22, 200 (2000)

Reich, M., et al., "Depression, quality of life and breast cancer: a review of the literature," Breast Cancer Res. Treat. 110, 9 (2008)

Short, P. F., et al., "Work disability associated with cancer survivorship and other chronic conditions," Psychooncology. 17, 91 (2008)

Spelten, E. R., et al., "Factors reported to influence the return to work of cancer survivors: a literature review," Psychooncology. 11, 124 (2002)

Spelten, E. R., et al., "Cancer, fatigue and the return of patients to work-a prospective cohort study," Eur. J. Cancer 39, 1562 (2003)

Taskila-Åbrandt et al., "Employment status of Finnisch cancer patients in 1997," Psycho-oncology. 14,221 (2005)

Taskila, T., et al., "Cancer survivors' received and needed social support from their work place and the occupational health services," Support Care Cancer. 14, 427 (2006)

Taskila, T. & Lindbohm, M. L., "Factors affecting cancer survivors' employment and work ability," Acta Oncol. 46, 446 (2007a)

Taskila et al. "Comperative study of work ability between cancer survivors and their referents". Eur. J. Cancer 43, 941 (2007b)

Verbeek and E. R. Spelten, "Work", in Handbook of cancer survivorship, edited by M. Feuerstein (Springer US, 2007)

Yabroff, K. R., et al., "Burden of illness in cancer survivors: findings from a population-based national sample," J. Natl. Cancer Inst. 96, 1322 (2004)
